# Effect of the use of eCG on the pregnancy rate in dairy cows treated with a prolonged proestrus protocol in the Ecuadorian Amazon

**DOI:** 10.1590/1984-3143-AR2025-0084

**Published:** 2026-02-02

**Authors:** Manuel Edelberto Ortega-Coello, Orlando Roberto Quinteros-Pozo, Pablo Roberto Marini, Gabriel Amílcar Bó

**Affiliations:** 1 Centro Latinoamericano de Estudios de Problemáticas Lecheras, Universidad Nacional de Rosario – UNR, Rosario, Santa Fe, Argentina; 2 Carrera del Investigador Científico – CIC, Universidad Nacional de Rosario, Rosario, Santa Fe, Argentina; 3 Universidad Estatal Península de Santa Elena, La Libertad, Santa Elena, Ecuador; 4 Facultad de Ciencias Veterinarias, Universidad Nacional de Rosario, Rosario, Santa Fe, Argentina; 5 Instituto de Reproducción Animal Córdoba – IRAC, Córdoba, Argentina; 6 Medicina Veterinaria, Instituto Académico Pedagógico de Ciencias Básicas y Aplicadas, Universidad Nacional de Villa María, Córdoba, Argentina

**Keywords:** Holstein cows, proestrus, eCG, follicular diameter, progesterone

## Abstract

The objective of this study was to evaluate the effect of eCG on the time of ovulation, preovulatory follicle diameter and pregnancy rate in Holstein cows with a prolonged proestrus protocol in the Ecuadorian Amazon. This research was conducted between October 2018 to October 2019. We used 447 multiparous cows, milked once a day, with an average milk production of 8 to 9 liters cow/day. Two treatments were used, T1 with the addition of eCG (206 cows); and T2 without the application of eCG (241 cows). The pregnancy rate was higher in cows that received eCG (59.2%, T1) than in cows that did not receive eCG (45.6% T2: P<0.05), but the presence or absence of estrus did not differ (P>0.05) among groups (T1: 70.4%), T2: 65.6%). The diameter of the dominant follicle at P4 device removal (T1: 9.5 ± 0.6 mm vs T2: 9.7 ± 0.7 mm), as well as at the time of artificial insemination (T1: 11.6 ± 0.7mm vs T2: 11.2 ± 1.1mm) was not different among groups (P>0.05). However, the diameter of the dominant preovulatory follicle was greater (P<0.05) in cows in T1 (13.5 ± 1.1 mm) than those in T2 (12.5 ± 1.6 mm), with a follicular growth rate of 1.3 ± 0.1 mm/day and 0.9 ± 0.1 mm/day, for T1, T2 respectively (P<0.05). The mean ovulation time from device removal was shorter for T1 (94.7 h; P<0.05) than for T2 (101.5 h). The diameter of the CL was greater (P<0.05) for T1 (20.8 ± 2.3 mm) than T2 (19.1 ± 3.6 mm), while plasma concentrations of P4 did not differ among groups (P>0.05). In conclusion, the addition of eCG to the J-Synch protocol increased the diameter of the preovulatory follicle and the resulting CL, which favored the pregnancy rate in cows in the Ecuadorian Amazon.

## Introduction

Artificial insemination (AI) in combination with heat detection is not used in the Amazon region, as it is difficult for farmers to observe and detect the signs of heat due to the characteristics of the terrain, the poor physical condition of the cows, and the long lactation period (Quinteros, 2009; Moyano, 2013). One way to solve this problem and increase the number of cows inseminated is to use protocols that allow ovulation to be synchronized through artificial insemination at a specific time (AITF; López et al., 2014). Baruselli et al. (2004) have shown that the use of the hormone eCG creates conditions that stimulate follicular growth and ovulation, which has a positive effect on cows with postpartum anestrus, especially those that have recently calved (postpartum period less than 60 days), animals in poor body condition, and animals with impaired dominant follicle growth (Baruselli et al., 2004; Bó et al., 2007). In addition, the application of eCG at the time of removal of the intravaginal P4 implant showed an increase in the concentration of P4 in the blood plasma.

Two trials were conducted with lactating dairy cows to compare a control protocol called “conventional” with ECP as an ovulation inducer and two protocols with prolonged proestrus (J-Synch). Both J-Synch 6 and J-Synch 7 were found to have similar pregnancy rates in lactating dairy cows (Macagno et al., 2022).

Two experiments were conducted in New Zealand to compare the reproductive response of lactating dairy cows with seasonal calving, diagnosed as anestrus by rectal palpation (Bryan et al., 2010). There were no significant differences between a 6- or 7-day P4 treatment protocol, and there were no interactions between 6/7 days and eCG treatment. However, the inclusion of eCG in a GnRH plus P4 synchronization program significantly increased the pregnancy rate at 7 days and 28 days and reduced the median days to conception. The results confirm that adding eCG to synchronization protocols for anestrus cows in seasonal calving dairy herds in New Zealand is valuable in getting more cows pregnant early in the breeding season. However, when this same protocol was evaluated in 1,421 lactating cows in a seasonal system in Ireland, administration of eCG at the time of removal of the P4 device did not result in a higher pregnancy rate for cows treated or untreated with eCG, respectively (Randi et al., 2018). The authors attributed the lack of difference in pregnancy rates to the fact that dairy cows in Ireland were generally in better nutritional condition than those in New Zealand.

In two experiments in Argentina (Bó et al., 2011), the effect of adding eCG to a protocol with a P4 device with GnRH on follicular dynamics and pregnancy rates in lactating Holstein cows in a mixed management system was evaluated, finding that the pregnancy rate was not different between cows treated or not treated with eCG. Ferreira et al. (2013) reported similar results in a study in Brazil. In another experiment also conducted in Brazil in a free stall system (Souza et al., 2009), pregnancy rates were higher in cows with lower body condition that were treated with eCG than in those that were not treated with eCG. In contrast, pregnancy rates did not differ in cows with optimal condition. Finally, the administration of eCG at a dose of 400 IU at the time of removal of the intravaginal device with P4 in dairy Normande cows and heifers located in moorland areas above 2800 meters above sea level in the municipality of Caldas, Colombia, treated with a conventional IATF protocol, had a positive impact on pregnancy rates (Ospina and Ramos, 2013).

While it is true that in recent years studies have been conducted in the Ecuadorian Amazon using estrus synchronization protocols, particularly the prolonged proestrus protocol (J-Synch) in dual-purpose cows and, to a lesser extent, in dairy cows. Therefore, it is important to evaluate this protocol with dairy breeds in an adverse climate such as the Amazon, with management practices adapted to the environment, which are very different from those carried out in temperate climates and in specialized cows that have genetic material that cannot express their potential.

The objective was to evaluate the effect of adding eCG on follicular diameter, blood progesterone concentration, and pregnancy rate in multiparous dairy cows in the Ecuadorian Amazon.

## Materials

### Animals and workplace

**Figure 1:**
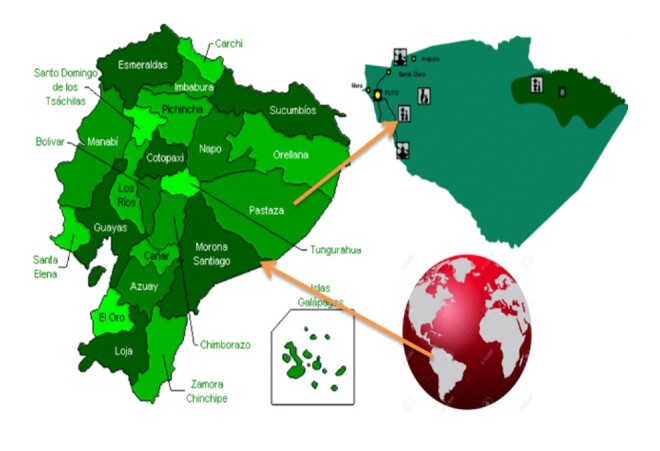
Political map of Ecuador—Province of Pastaza.

The work was carried out in production units located in the dairy basin of the Province of Pastaza, Canton of Pastaza, Parishes: Veracruz, Diez de Agosto, and El Triunfo, geographically located in the center of the Ecuadorian Amazon Region, between the geographical coordinates 1° 10 South Latitude and 78° 10 West Longitude, 2° 35 South Latitude and 76° 40 West Longitude, with a humid tropical climate, temperatures averaging 25 °C (15-30 °C), and rainfall of 4000-5000 mm/year. Average annual humidity is 90 to 100%. Its topography is characterized by gently rolling hills without steep slopes, distributed across large natural plateaus. The altitude varies between 900 and 1100 meters above sea level (Figure 1).

A total of 447 multiparous Holstein cows in production were used, fed using a tethered grazing system (Carrera Durazno et al., 2015) with an average milk production of 8.0 liters (cow/day) milked once a day in the presence of their calves, in the same place where they graze under extreme weather conditions, outdoors, with rain most of the year. The cows were selected for the presence of follicles > 8 mm in diameter or a CL in their ovaries, detected by ultrasonography (Ibex veterinary ultrasound scanner – pro with 5 MHz linear probe in B mode; 106/206; 51% with CL in T1 and 133/241; 55% with CL in T2). The cows used ranged from 90 to 150 days open (119 ± 23.5 days), with ages ranging from 34 to 65 months (52.6 ± 8.8 months), body condition score of 2.4 ± 0.1 (range 2.25 to 2.75 on a scale of 1-5; Edmonson et al., (1989) and a live weight between 419 ± 19 kg (range 400 and 450 kg).

The farms where the trials were conducted have an average area of 50 hectares, consisting of natural pastures typical of the area. The cows received a supplement (150 g of mineral salts + 1 kg of balanced feed + 1 kg of wheat bran) per day two or three times a week during milking.

## Methods

### Treatments and experimental design

The cows were randomly divided into two groups:

T1: J-Synch + eCG (206 cows)

T2: J-Synch (241 cows)

The distribution of treatments was completely random, with eight replicates per treatment as follows: T1: 25, 26, 25, 26, 25, 26, 26, 27 (206); T2: 30, 30, 30, 30, 30, 30, 30, 31 (241). In this trial for both treatments, on Day 0, 2 mg of BE (Gonadiol, Zoetis, Ecuador) was administered intramuscularly (i.m.) together with a 0.5 g DIB intravaginal device of P4 (Zoetis, Ecuador).

For T1, on Day 6, the intravaginal device was removed along with the i.m. administration of PGF2α (500 μg of Cloprostenol, Ciclase, Zoetis) plus the i.m. administration of 400 IU of eCG (Novormón 5000, Zoetis). On the same day, a paint marker was applied to the base of the tail as an indicator to identify whether there was heat prior to IATF. Of this group of cows, only those that showed heat (135 cows) within 60 hours after removal of the device were inseminated exactly 60 hours later and underwent follicular measurement by ultrasonography. Cows that did not show estrus 60 hours after removal of the intravaginal device (71 cows) were inseminated at 72 hours using IATF in conjunction with intramuscular administration of 100 µg of gonadorelin acetate (GnRH, Gonasyn GDR, Zoetis), in addition to an ultrasound assessment to determine the diameter of the preovulatory follicle at IATF.

In the case of T2, on Day 6, the intravaginal device was removed together with the intramuscular administration of PGF2α, but without the administration of eCG. On this day, a paint marker was applied to the base of the tail as an indicator to identify whether there was heat prior to IATF. As in the previous group, cows that showed estrus (104 cows) within 60 hours after removal of the intravaginal device were inseminated by AI and underwent follicular measurement by ultrasound. Those that did not show estrus (98 cows) were inseminated using IATF in conjunction with intramuscular administration of GnRH, in addition to an ultrasound assessment to determine the diameter of the preovulatory follicle at insemination.

For IATF, frozen/thawed semen was used, employing the technique described by Bernardi et al. (2011), from a single bull of proven fertility, although it was also analyzed before use and the cows were inseminated by the same technician.

### Treatment schedule

**Figure 2:**
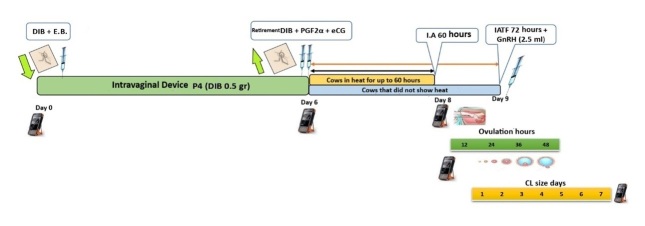
T1. J-Synch + eCG. Cows in heat AI 60 h, without heat IATF 72 h + GnRH. **Caption:** E.B. (estradiol benzoate), PGF2α (prostaglandin), GnRH (gonadotropins), eCG (equine chorionic gonadotropin), IATF (fixed-time artificial insemination), DIB (intravaginal device with 0.5 g P4).

Figure 2 illustrates the estrus synchronization protocol with prolonged proestrus called J-Synch (Yánez-Avalos et al., 2021), which is modified by adding eCG and performing FTAI at 60 hours without estrus, plus the application of eCG at 72 hours after removal of the intravaginal device.

**Figure 3:**
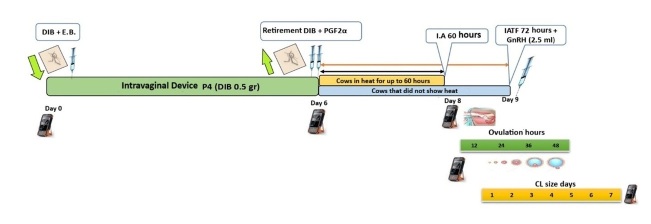
T2. J-Synch. Con Celo IA 60 h, sin Celo IATF 72 h + GnRH. **Caption:** E.B. (estradiol benzoate), PGF2α (prostaglandin), GnRH (gonadotropins), eCG (equine chorionic gonadotropin), IATF (fixed-time artificial insemination), DIB (intravaginal device with 0.5 g P4).

In Figure 3, the estrus synchronization protocol with prolonged proestrus called J-Synch (Yánez-Avalos et al., 2021) is illustrated, where FTAI is performed at 60 hours without estrus, plus the administration of eCG at 72 hours after removal of the intravaginal device.

### Ultrasonography

Ovarian status was assessed on Day 0 by determining the presence of follicles > 8 mm in diameter or CL, prior to placement of the intravaginal device with P4 (Ibex Pro and Lyte® ultrasound scanner, USA, with 5.0 MHz linear transducer). On Day 6, after removal of the intravaginal device, the second follicular measurement was performed on all cows. After removal of the intravaginal device, follicular size was obtained by ultrasonography. For this trial, a sample of the total number of cows used was selected completely at random: T1 (33/206 cows) and T2 (20/241 cows). Ultrasonography was then performed 12 hours after IATF, Then, animals that had not yet ovulated underwent another ultrasound scan 24 hours later, and animals that had not yet ovulated at this point were subjectively determined to have ovulated > 24 hours after IATF. On day 7 post-IATF, the size of the CL was measured. To do this, two measurements (height – width in mm) were always taken of each structure (CL or follicle) and averaged to obtain a more approximate measurement (Perry and Cushman, 2016). Pregnancy diagnosis was determined by ultrasonography between 35 and 40 days after IATF.

### Observation of estrus

Estrus was recorded using the visual method and with a paint marker (Markings Tick, Divasa-Farmavic, Spain) by placing a strip 20 to 30 cm long by 5 cm wide at the base of the cow's tail (Giraldo, 2007). Considering that the animals were kept in a grazing system, any animal that showed more than 50% loss of color 48 to 60 hours after removal of the device, due to mounting by another animal or the presence of crystalline vaginal secretion on the tail, was in heat. Visualization began after removal of the intravaginal device three times a day: morning, noon, and afternoon (Days 7, 8, and 9) until prior to IATF.

### Blood sampling on day 7 post-IATF and P4 analysis

The cows were tied and immobilized, after cleaning and disinfecting the base of the tail, by puncture with needles and vacutainer tubes without anticoagulant, blood samples were extracted from the median coccygeal vein in volumes of 8 mL. The samples were stored at 4 °C for 4-6 hours from extraction until arrival at the Laboratory of the Amazon Postgraduate Research and Conservation Center (CIPCA), where the samples were centrifuged at 3000 rpm for 20 minutes to separate the blood serum, which was frozen at -20 °C until further analysis (de Castro et al., 2004). A total of 80 samples were used for the analysis of serum P4 concentrations, which are the cows that underwent ultrasound for follicular dynamics and ovulation monitoring: (T1 = 40 samples) and (T2 = 40 samples), which were analyzed individually in duplicate using P4 kits (iChroma™P4 CFPC - 21– Korea). The fluorescence immunoassay (FIA) technique was used for the quantitative determination of P4 in serum. The test uses a competitive immunoassay method. In this method, the analyte in the sample binds to the fluorescently labeled (FL) detection antibody in the detection buffer to form the complex as a sample mixture. This complex is loaded to migrate to the nitrocellulose matrix, where the covalent pair of P4 and bovine serum albumin (BSA) are immobilized and interfere with the binding of the analyte and the fluorescently labeled antibody (FL). If there are more analytes in the sample, fewer detection antibodies accumulate, resulting in less fluorescence signal (Abraham et al., 1972). In terms of sensitivity, the minimum detectable concentration of P4 that can be distinguished in a working range is: (0.127 to 127.2 nmol/L and 0.4 to 40 ng/mL with a 99% confidence limit. The intra-assay coefficient of variation (CV) was: 5.2% (low) and 6.2% (high).

### Variables analyzed

The following variables were analyzed in the study (Table 1).

**Table 1:** Variables evaluated in the experiment.

**Variables**	**Range / Unit**
Ovarian status on Day 0	presence of CL or follicles >8 mm
Heat expression	Presence or absence
Follicular diameter	mm
Time of ovulation	h after device removal
CL size on Day 7 post-IATF	mm
Serum P4 concentration	ng/mL
Pregnancy rate	Pregnant or empty

Table 1 shows the variables that were evaluated in the experiment, as well as the ranges or units in which they are expressed throughout the document.

### Statistical analysis of the data

For each continuous variable studied, the arithmetic mean, and standard error (SE) were estimated. The data were analyzed using analysis of variance (ANOVA) with a classification criterion and Tukey-Kramer HSD multiple comparison tests (P<0.05). For the analysis of pregnancy rates and estrus expression, a multiple logistic regression model was fitted, as this considers that the response variable (Confirmation) is binary.

The following logistic regression model was proposed. Its objective was to analyze the relationship between CL size, follicle size on Day 6, the treatment applied, and the time of IATF with pregnancy confirmation.


$$Logit\left(\pi_{i}\right)=\log\left(\frac{\pi_{i}}{1-\pi_{i}}\right)=\beta_{0}+\beta_{1}CL_{i}+\beta_{2}F6_{i}+\beta_{3}Trat_{i}+\beta_{4}Hora_{i}$$ (1)

We evaluated whether it was necessary to add interactions between the variables in the model. Each row of the following table contrasts the following hypotheses using the likelihood ratio test:


$$H_{0})\;\beta j\;=\;0\;$$ (2)

$$H_{1})\;\beta j\;\neq \;0\;$$ (3)

Given the sample evidence and with a significance level of 5%, we can affirm that it is not necessary to add any of the interactions to the model. The model was interpreted using estimated odds ratios, together with their confidence intervals. The analysis was performed using R 4.0.2. software.

### Ethical handling of animals

Data was collected on the animals in the study in their environment during regular zootechnical activities. These practices were non-invasive, as described by Yánez-Avalos et al. (2021). Even so, the animals were handled in such a way that the minimal manipulation did not cause them stress. As production animals, they regularly enter the handling chute for both health and reproductive checks, at this time, data on the treatments was collected, in accordance with Resolution XX of the Ecuadorian Agency for Phytosanitary and Zoosanitary Regulation and Control (Ecuador, 2021), Article 1, which establishes the regulations for the formation, approval, and monitoring of Ethics Committees for animal research in Ecuador, and Article 3. - Scope of application, it does not establish that the authorization of an Ethics Committee is mandatory for the collection of data on regular and routine zootechnical treatments or procedures in a production system or in a reproductive management program on the farm.

## Results

### Ovarian status on day zero (0) at the start of treatment

Table 2 shows the effect of the presence of CL or superficial anestrus (follicle >8 mm) measured on day 0 (start of protocol) on the pregnancy rate. The pregnancy rate did not differ between T1 and T2 for cows that showed a CL, while in cows that showed superficial anestrus, the pregnancy rate was higher (P<0.05) in T1 than in T2. In addition, the overall pregnancy rate was higher (P<0.05) in cows that presented CL than in cows that exhibited superficial anestrus.

**Table 2:** Effect of cyclicity on day 0 on the pregnancy rate.

**Treatments**	**No. animals**	**CL Day 0**	**Pregnancy%**	**F.O Day 0**	**Pregnancy%**
**T1**	206	106	67.9 ^a^ (72/106)	100	50.0 ^a^ (50/100)
**T2**	241	131	60.3 ^a^ (79/131)	110	28.2 ^b (^31/110)
**TOTAL**	**447**	**237**	**63.7 ^x^ (151/237)**	**210**	**47.0 ^y^ (81/210)**

ab Means in the same column with different superscripts differ statistically (P<0.05); ^xy^Means in the same row with different superscripts differ statistically (P<0.05).

### Heat expression

In this study, it was observed that 67.8% of cows presented heat within 60 hours after removal of the intravaginal device.

Table 3 shows that the percentage of animals in heat in T1 was 70.4%, while in T2 it was 65.6%. However, these differences were not statistically significant (P=0.324).

**Table 3:** Presence or absence of heat symptoms.

**Treatment**	**No. of animals**	**In heat**	**Heat rate %**
**T1**	206	145/206	70.4
**T2**	241	158/241	65.6
**Total**	**447**	**303/447**	**67.8**

The percentages do not differ (P>0.05).

In this same context, when relating the presence of heat and pregnancy rate by treatment (Table 4).

**Table 4:** Presence of estrus and pregnancy rate by treatment.

**Treatments**	**No. of animals**	**In estrus**	**% Pregnancy**	**Not in estrus**	**% Pregnancy**
**T1**	206	89/145	61.4 ^a^	33/61	54.1 ^a^
**T2**	241	75/158	47.5 ^a^	34/83	41.0 ^a^
**TOTAL**	447	164/303	54.1 ^a^	67/144	46.5 ^b^

ab Means in the same row with different superscripts differ statistically (P<0.05).

Table 4 shows that in T1, among cows that showed estrus (J-Synch with eCG + AI 60 h), the pregnancy rate was 61.4%, while among cows that did not show estrus (J-Synch with eCG + I.ATF 72 h), it was 54.1%. Similarly, in T2, among cows that showed estrus (J-Synch without eCG + AI 60 h), the pregnancy rate was 47.5%, and among cows that did not show estrus (J-Synch without eCG + IATF 72 h), it was 41.0%, with no statistical differences (P>0.05).

Diameter of the dominant ovulatory follicle at the time of device removal, at the time of IATF, and prior to ovulation

Table 5 shows the size of the dominant follicle at the time of removal of the intravaginal device and at the time of IATF, which do not differ statistically between the groups. However, the preovulatory follicle at the end of proestrus was larger for T1 than for T2. The differences are statistically significant.

**Table 5:** Means and standard errors of follicular size (mm) in T1 and T2

**Treatment**	**N**	**At removal (Day 6)**	**At IATF** **(Day 8)**	**End of proestrus**	**Growth rate (mm/day)**
**T1**	33	9.5 ± 0.6 ^a^	11.6 ± 0.7 ^b^	13.5 ± 1.1 ^a^	1.3 ± 0.1 ^a^
**T2**	20	9.7 ± 0.7 ^a^	11.2 ± 1.1 ^b^	12.5 ± 1.6 ^b^	0.9 ± 0.1 ^b^

ab Means with different superscripts differ statistically (P<0.05).

### Time of ovulation

The mean interval of ovulation from the removal of the intravaginal device is shown in (Table 6), being greater for T2 than for T1, showing statistically significant differences (P<0.05) between treatments.

**Table 6:** Ovulation rate and interval from removal of the device with P4 to ovulation.

**Treatments**	**No. of animals**	**Hours to ovulation**	**Ovulation rate %**
**T1**	32/33	94.7 ± 7.2 ^a^	96.9
**T2**	18/20	101.5 ± 7.1^b^	90.0
**Total**	**50/53**	**98.1 ± 7.2**	**94.3**

ab Means with different superscripts differ statistically (P<0.05).

**Figure 4:**
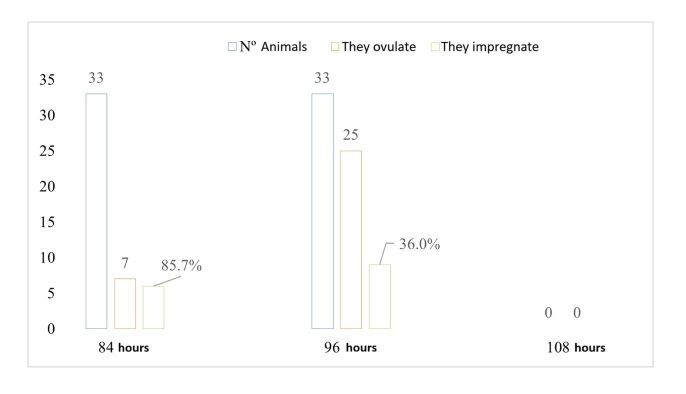
Ovulation response and pregnancy rate recorded as a percentage at 84, 96, and 108 hours after IATF for cows in T1.

**Figure 5:**
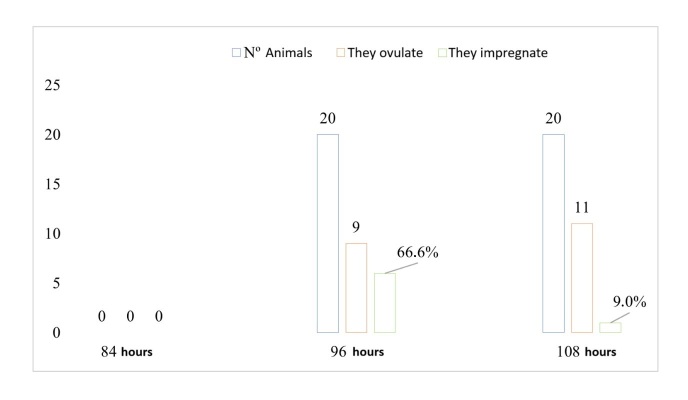
Ovulation response and pregnancy rate, recorded as a percentage, at 84, 96, and 108 hours after IATF for cows in T2.

Figures 4 and 5 shows the number of cows evaluated by ultrasonography that ovulated in each of the treatments, as well as the pregnancy rate based on the time of ovulation. T1 had a higher number of cows that ovulated at 96 h with a pregnancy rate of 36.0% than those that ovulated at 84 h (P<0.05), which had a pregnancy rate of 85.7%.

In T2, the cows ovulated later, with a higher number of cows ovulating up to 108 hours, with a pregnancy rate of 9.1%, than those that ovulated at 96 hours, which had a pregnancy rate of 66.7%.

### CL size 7 days after IATF

Table 7 refers to the CL diameter measured on day 7 post-IATF, so we can see that it differed significantly between treatments (P<0.05).

**Table 7:** CL size on Day 7 post-IATF.

**Treatments**	**No. of animals**	**CL diameter (mm)**
**T1**	206	20.8 ± 2.3 ^a^
**T2**	241	19.0 ± 2.6 ^b^
**Total**	**447**	**19.9 ± 2.9**

ab Means with different superscripts differ statistically (P<0.05).

### Serum P4 concentrations on day 7 after IATF

Table 8 shows the CL diameter values (only for sampled cows) and serum P4 concentrations in the treatments: (J-Synch with eCG and J-Synch without eCG) in the subgroups of 40 animals from which blood samples were taken. No statistically significant differences (P>0.05) were found between treatments.

**Table 8:** CL diameter and serum P4 concentrations.

**Treatments**	**No. of animals**	**CL diameter (mm)**	**P4 concentration (ng/mL)**
**T1**	40	20.5 ± 2.0	10.2 ± 2.6 ^a^
**T2**	40	19.4 ± 2.6	9.1 ± 2.5 ^a^
**TOTAL**	**80**	**20.1 ± 2.3**	**9.6 ± 2.5**

The means do not differ (P>0.05).

### Pregnancy rate

Regardless of the treatments used, of the 447 cows inseminated, 232 cows became pregnant, representing 51.9%.

Table 9 shows that the pregnancy rate was higher (P<0.05) in cows treated with eCG (T1) than in those not treated with eCG (T2). The values differ statistically between treatments.

**Table 9:** Pregnancy rate in dairy cows treated with the J-Synch protocol.

**Treatment**	**No. of animals**	**Pregnant**	**Pregnancy rate (%)**
**T1**	206	122/206	59.2 ^a^
**T2**	241	110/241	45.6 ^b^
**Total**	**447**	**232/447**	**51.9**

ab Means with different superscripts differ statistically (P<0.05).

### Logistic regression model

The logistic regression model, which aimed to analyze the relationship between CL size, follicle size on Day 6, and IATF time with pregnancy confirmation, determined that CL size, follicle size on Day 6, and IATF time were significantly associated with pregnancy (Table 10).

**Table 10:** Estimated coefficients and Wald test.

Parameter	gl	Estimate	Standard Error	P-value
Intercept	1	-26.87	2.99	<0.001
CL	1	1.08	0.10	<0.001
F 6	1	0.56	0.21	0.009
Time	1	-0.67	0.30	0.027

If we analyze the odds ratios (Table 11), we can say that, controlling for other variables, the chance of a cow becoming pregnant triples when the CL increases by one millimeter. Furthermore, this chance improves by 74% when the follicle size on Day 6 grows by one millimeter. Finally, controlling for other variables, the likelihood of the animal becoming pregnant decreases by 50% when IATF is applied at 72 h, compared to 60 h. That is, when she does not show estrus during the first 60 h after the device is removed.

**Table 11:** Odds ratio estimates and confidence intervals.

**Odds ratios**	**Estimate**	**Confidence interval**	
CL	2.95	2.41	3.61
F6	1.74	1.15	2.64
Time 72 h vs. 60 h	0.51	0.29	0.93
Whit eCG vs. Without eCG	1.55	0.85	2.82

## Discussion

In the Ecuadorian Amazon, López Parra et al. (2024) showed results using the J-Synch protocol in dual-purpose cows and compared it with conventional protocols (BE and ECP), obtaining encouraging results. Under Amazonian production conditions, the J-Synch protocol has been modified and variants added, such as the addition of eCG and the timing of IATF in the presence or absence of estrus. The addition of eCG has sought to improve the function of the ovulatory follicle by stimulating its growth and activity in the period between the removal of the P4 device and ovulation (Menchaca et al., 2019). In addition, the prolongation of proestrus generates a longer exposure to circulating serum concentrations of estradiol produced by the dominant follicle, favoring the uterine environment, and improving fertility (Bridges et al., 2014; de la Mata et al., 2018).

When performing an ultrasound scan on Day 0 of the synchronization program (Table 2), the presence of CL is synonymous with cyclic cows. The pregnancy rate did not differ between cyclic cows treated or not treated with eCG. On the contrary, statistical differences were found among cows with superficial anestrus, indicating that the addition of eCG increases the pregnancy rate, especially in cows in anestrus. These data do not agree with those shown by Yánez (2022), since in his study the cows were clearly in better condition, as 70% of the cows had a CL, with no significant difference between cows with CL (52.3%) and cows with follicles >8 mm (52.0%). However, Menchaca et al. (2006) found higher pregnancy rates in cows that were in anestrus and were treated with eCG, with no significant differences in cows with a CL, data that are like those in the thesis in question.

Dairy cows in the Amazon region of Ecuador have a short estrus duration and tend to express it during the night. In this study, a high percentage of estrus expression was observed (Table 3). Although they had lower values than those reported by López (2017), they were above those reported by Yánez (2022). Macagno et al. (2022), who evaluated treatments for ovulation synchronization in lactating dairy cows—a conventional protocol and two prolonged proestrus protocols (J-Synch)—found that the overall expression of estrus was like this study.

The interval to ovulation (Table 6) for cows treated with eCG was higher than the results shown by Yánez (2022) in dual-purpose cows in the Amazon, indicating that perhaps the conditions of dairy cows in the Amazon cause them to ovulate earlier, so the timing of AI should be brought forward to 60-66 days after removal of the P4 device.

About the ovulation rate (Table 6), although the differences were not significant in this study, they were higher for cows treated with eCG than for those not treated with eCG, similar to the findings reported by Núñez-Olivera et al. (2020) and Yánez (2022), confirming that the effect of eCG in stimulating follicular growth results in a higher ovulation rate and, consequently, a higher pregnancy rate.

The results of the diameters of the dominant ovulatory follicles found in this study (Table 5) showed a larger diameter for cows treated with eCG compared to those not treated with eCG. The larger size of the preovulatory follicle was due to a higher growth rate of the dominant follicle induced by eCG, which resulted in a higher pregnancy rate. On the other hand, Yánez (2022) in Brown Swiss cows in Ecuador did not find greater growth of the dominant follicle in cows treated with eCG and, therefore, did not find significant differences in pregnancy rates.

The CL diameter (Table 7) achieved in this experiment differs from other studies conducted in the Ecuadorian Amazon, where Yánez (2022) showed 21.7 ± 0.2 mm for J-Synch without eCG and 22.7 ± 0.2 mm for the group with eCG in Brown Swiss cows. Perhaps the cows in Yanez's experiment were in better condition than the cows in the present study, which is why the CLs were larger on average. López (2017) reported an average CL diameter of 25.4 mm in dual-purpose cows treated with the J-Synch protocol, which, although no significant differences were found, was larger than that obtained with conventional protocols using BE or ECP as ovulation inducers.

About P4 concentrations (Table 8), there were no statistically significant differences between treatments. This contrasts with Yánez (2022), who obtained higher values (9.3 ± 0.2 ng/mL) for Brown Swiss cows in the Ecuadorian Amazon treated with eCG than for those not treated with eCG (8.5 ± 0.2 ng/mL). Similar results were reported by Núñez-Olivera et al. (2020), in which treatment with eCG showed higher concentrations of P4 compared to treatment without eCG. In this study, there was a trend, but it could not be confirmed that eCG stimulates the ovulatory follicle, resulting in a larger CL and higher concentrations of P4.

Table 10 shows the higher pregnancy rate in cows treated with eCG. These results confirm that eCG is a tool that can be used to improve pregnancy rates in dairy cows under the management conditions of the Ecuadorian Amazon. Considering (Table 11) the size of the CL, the size of the follicle on Day 6, and the timing of IATF to improve pregnancy

Yánez (2022), using dual-purpose cows, found a positive effect from the use of eCG. Perhaps the main reason for this discrepancy can be explained by the results of another experiment also conducted in Brazil, in a free stall system (Souza et al., 2009). In this experiment, the addition of eCG also did not improve the pregnancy rate in lactating cows; however, when cows with lower body condition scores (<2.75) were considered, these were higher in those treated with eCG (38.0%) than in those not treated with eCG (15.2%). This confirms that eCG will improve pregnancy rates in situations or animals where management and nutritional conditions affect the growth of the dominant preovulatory follicle.

It can be said that animals that show signs of estrus before being IATF result in higher pregnancy rates due to greater follicular diameter, higher ovulation rate, and greater CL development with greater P4 secretory capacity during the luteal phase (Perry et al., 2005, 2007; Sá et al., 2011).

In this thesis, the group of cows that received the J-Synch treatment with eCG had a longer proestrus duration, tended to have a higher follicular growth rate, a higher rate of estrus expression, and developed a larger CL that produced higher circulating P4 concentrations after ovulation, favoring pregnancy rates. In addition, when the cows from the two treatments were combined, those that showed signs of estrus had a higher pregnancy rate than those that did not show estrus, as has been reported in other studies (Cedeño et al., 2019, 2021; Tschopp and Bó, 2022; Tschopp et al., 2022).

The study generated necessary and original information for pasture-based milk production in the Ecuadorian Amazon, which is a unique and system in the world. There are no previous reports in Ecuador of reproductive responses to the inclusion of eCG in the prolonged proestrus protocol in Holstein dairy cows.

## Conclusions

The results obtained allow us to conclude that treatment with eCG resulted in a preovulatory follicle and a larger CL, and these results were reflected in a higher pregnancy rate at IATF. In addition, cows that received eCG had a shorter interval to ovulation than cows that did not receive eCG. Cows that showed estrus had a higher pregnancy rate than those that did not show estrus.
